# Arthroscopic Assisted Turf Toe Reconstruction With Knotless Suture Anchors and Suture Tape Augmentation

**DOI:** 10.1002/atn2.70196

**Published:** 2026-07-28

**Authors:** Nicholas K. Pappa, Kyle S. Ardavanis, Tobin Eckel, Kevin D. Martin

**Affiliations:** ^1^ Department of Orthopaedic Surgery The Ohio State University Wexner Medical Center Columbus Ohio U.S.A.; ^2^ Department of Orthopaedic Surgery Irwin Army Community Hospital Fort Riley Kansas U.S.A.

## Abstract

Turf toe is a hallux metatarsophalangeal joint (MTPJ) injury seen in sports like football and soccer where the foot experiences axial load while fixed to the ground with the great toe hyperextended at the MTPJ. Depending on severity, turf toe can be quite devastating in athletes and can require operative intervention. Here, we describe an arthroscopic approach to MTPJ to allow direct visualization and evacuation of joint hematoma as well as the utilization of all suture knotless suture anchors to eliminate knots and reduce suture burden, reduce soft tissue irritation, and accelerate recovery after surgery. We report our technique for arthroscopic assisted, capsular reconstruction of the great toe MTPJ with all knotless suture anchors and suture tape augmentation.

## Graphical abstract

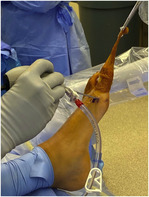

**VIDEO 1:** Left foot ‐ This video shows an arthroscopic approach to metatarsophalangeal joint to allow direct visualization and evacuation of joint hematoma as well as the utilization of all suture knotless suture anchors and suture tape augmentation to reconstruct the great toe metatarsophalangeal joint after turf toe injury. Video content can be viewed at https://doi.org/10.1002/atn2.70196.

Turf toe is an hyperextension injury of the metatarsophalangeal joint (MTPJ) of the great toe and associated plantar plate and sesamoid complex.^1^, ^2^ This injury is seen in high level athletes participating in contact sports like football and soccer. Severity of turf toe can range from sprain to complete tear of plantar plate with associated sesamoid injury and/or traumatic bunion.^3^, ^4^ Treatment is based on severity including non‐operative treatment versus surgery for complete tears in high level athletes or chronic injuries refractory to conservative management.^5^, ^6^

Operative management for turf toe can be indicated when there is large MTPJ capsular disruption, MTPJ gross instability, traumatic or progressive hallux valgus, presence of loose bodies or cartilage lesions, or diastasis, retraction, or fracture of the hallux sesamoids.^7^, ^8^, ^9^ Operative techniques include direct end to end repair with nonabsorbable suture, plantar plate advancement via bone tunnels or suture anchors, and concomitant procedures such as sesamoidectomies and/or McBride procedures for associated sesamoid injury and traumatic bunion.^2^ We report our technique to surgically address turf toe utilizing both arthroscopy and open approach to reconstruct the MTPJ and soft tissue with all knotless suture anchors and augment the MTPJ capsule with suture tape.

## SURGICAL TECHNIQUE

### Positioning and Setup

**FIGURE 1:**
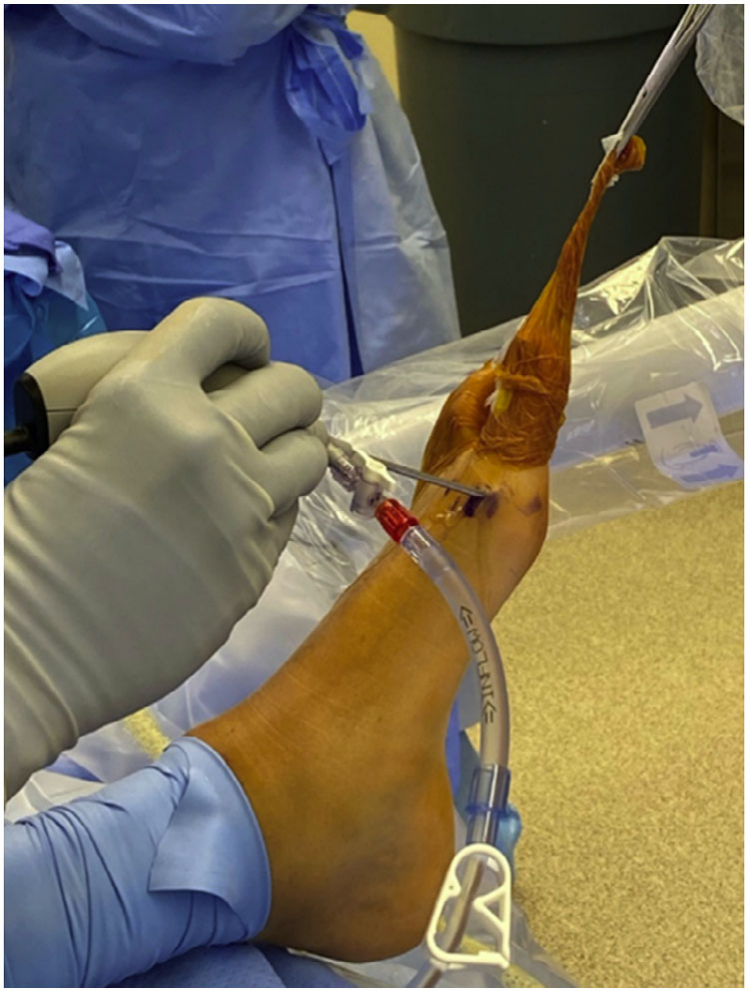
Great toe metatarsophalangeal arthroscopy set up with patient lying supine on the left foot. Kocher clamped to antimicrobial drape of hallux which is attached to external positioning arm for joint distraction on ipsilateral side of operative extremity. Dorsal medial and lateral portal incisions are created followed by insertion of nanoscope and shaver.

Patient is placed in supine position on operative table with small bump under ipsilateral hip of operative foot. External positioning arm is placed on ipsilateral side of operative extremity at distal aspect of bed rail. Thigh tourniquet is applied to operative extremity. Operative extremity is prepped and draped in standard manner. The hallux is wrapped separately with 2 layers of antimicrobial drapes. Then, a Kocher clamp is clamped to drape and attached to external positioning arm for joint distraction (Figure 1; Video 1).

### Great Toe MTPJ Arthroscopy

**FIGURE 2:**
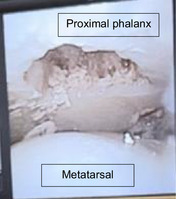
Arthroscopic visualization of great toe metatarsophalangeal joint of the left foot while under joint distraction. The metatarsal head and proximal phalanx are viewed within the middle of the joint. A significant chondral defect is noted at the articular surface of the proximal phalanx.

Prior to placing great toe in joint distraction apparatus, anatomic structures are outlined with sterile marker. Extensor hallucis longus tendon and great toe MTPJ are outlined. Dorsal medial and lateral portals are marked 5 mm from extensor hallucis longus tendon on each side. Great toe is placed in joint distractor as described above. Operative leg is elevated and exsanguinated followed by inflation of tourniquet to 250 mm Hg. The nick and spread technique is utilized to access the great toe MTPJ using dorsal medial and dorsal lateral portal sites. A 1.5 mm arthroscope (NanoScope; Arthrex, Naples FL) is introduced into the joint followed by a 2.5 mm shaver. The articular surface and capsule of the MTPJ is then well visualized with the NanoScope (Figure 2; Video 0:30). The 2.5 mm shaver is used to document and debride any soft tissue or cartilaginous lesions. Additionally, the water outflow flushes out the matrix metalloproteinases (MMPs) and inflammatory cytokines from the joint often associated with cartilage degradation.^10^ The 2 portal incisions are irrigated then closed with simple nylon stitch prior to proceeding to the open turf toe repair.

### Open Medial Approach

Longitudinal incision is made over direct medial aspect of great toe centered at level of MTPJ (Video 1:25). Meticulous dissection carried down to MTPJ with caution to avoid nearby dorsal cutaneous nerve. Soft tissues are retracted and medial capsule is identified, often with a rent/defect. Small periosteal flaps are then elevated dorsally to expose metatarsal head and proximal phalanx. Dissection is continued medially and plantarly at MTPJ to fully expose the medial eminence and the disrupted sesamoid collateral ligament and sesamoid sling which contributes to the plantar capsule at the plantar aspect of the base of the proximal phalanx.

### Sesamoid Complex and Capsular Reconstruction

**FIGURE 3:**
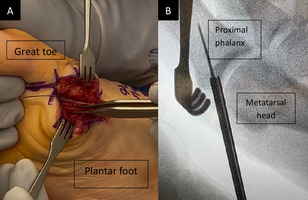
Open plantar plate reconstruction of the right foot. (A) Plantar plate exposure at level of great toe MTPJ prior to plantar plate reconstruction. (B) Drilling into proximal phalanx prior to placement of suture anchor with adequate position confirmed on fluoroscopy. (MTPJ, metatarsophalangeal joint.)

The medial eminence and medial base of proximal phalanx are prepared for reconstruction (Video 2:14). Distally the medial base of the proximal phalanx is drilled at isometric point for suture tape augmentation (Internal Brace; Arthrex, Naples FL). Next, the corresponding location for the suture tape is drilled at the medial eminence 6 mm from the articular surface (Video 2:49). Then, a knotless anchor (FiberTak; Arthrex, Naples FL) is placed 3 mm proximal from the articular margin of the metatarsal head; this suture is used to repair the medial capsule with a horizontal pattern. Next, the plantar aspect of the proximal phalanx is prepped, and an additional knotless anchor is placed at the plantar plate insertion; fluoroscopy can be used to confirm positioning (Figure 3). The suture is then passed through the tibial sesamoid sling, and with the toe in 20 degrees on plantarflexion, it is tensioned pulling the sesamoid distally restoring length.

### Angular Deformity Correction of Great Toe

**FIGURE 4:**
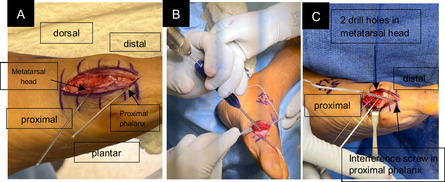
Drilling and placement of suture anchors and suture tape within metatarsal head and proximal phalanx of the left foot. (A) Drill holes for suture tape are created in metatarsal head and proximal phalanx. (B) Drill hole for suture anchor created in metatarsal head proximal to suture tape drill hole. (C) Interference screw with suture tape inserted into proximal phalanx.

**FIGURE 5:**
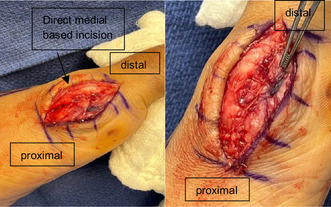
Capsule and soft tissue closure of MTPJ of the left foot. (A) Final appearance of suture tape grossly after passage of tape through capsule and tensioning of soft tissue. (B) Reinforcement of repair with 2‐0 Vicryl in running locking stitch fashion within more superficial soft tissue. (MTPJ, metatarsophalangeal joint.)

Suture tape is then placed into proximal phalanx secured with an interference screw (Swivel Lock; Arthrex, Naples FL). The suture tape is passed with free needle through the deep capsule but over top of the capsular tissue outside the joint (Video 3:16). The suture tape is passed a second time through the proximal capsule to strengthen the repair and ensure it is extra‐capsular. The suture tape is then passed through the drill hole of the metatarsal head with a nitinol passing wire (Figure 4). With the toe in neutral position, the suture tape is tensioned accordingly without overtightening to allow for adequate range of motion. Another interference screw is placed into the metatarsal head. After doing so, the angular deformity should be corrected with the toe remaining in neutral position (Video 3:50). 2‐0 Vicryl is placed in running locking stich fashion in the more superficial soft tissue to prevent suture irritation (Figure 5; Video 4:11). The wound is irrigated and closed with 3‐0 Vicryl for subcutaneous tissue and 3‐0 nylon for skin. Petrolatum gauze is applied to all incisions and a detailed great toe spica dressing is applied wrapped in plantar medial direction with elastic bandage.

### Postoperative Plan

Patient is non‐weightbearing for 4 weeks, with first 2 weeks in splint followed by 2 weeks in boot. Patient will take aspirin 81 mg BID for 2 weeks for deep vein thrombosis prophylaxis. At postoperative week 4, patient begins progressive weightbearing in boot with toe strapped in neutral position. Patient transitions from boot to carbon fiber insert at week 8 and begins active great toe motion with a soft silicone toe spacer to prevent valgus drift. Patient begins proprioception exercises and walking to jogging progression from weeks 8 to 12. At week 12, patient progresses to running program with physical therapy. Return to play without restrictions is usually expected at 6 to 9 months.

## DISCUSSION

This combined arthroscopic and open technique has its benefits. Arthroscopy allows evaluation of MCPJ articular surface of great toe through 2 portal incisions, decreasing the soft tissue morbidity. Visualization is further improved with indirect joint distraction. Additionally, utilizing inflow and outflow of saline through the MTPJ via arthroscopy allows inflammatory cytokines and MMPs to be washed out of the joint. This is important as it has been shown that MMPs and cytokines within a joint after trauma can lead to cartilage destruction.^10^ A major advantage of the all knotless suture anchors is the lack of knots in the foot which theoretically reduces any pain associated with knots and possible user error of knot tying. Additionally, there is limited space for implants in the metatarsals and phalanges of the foot. Fortunately, knotless suture anchors and suture tape have small insertional diameters. This property allows them to minimize over overcrowding the available bone stock while still providing significant strength and stability. However, it is important to note the possibility of overtightening the soft tissues of the MTPJ causing an alteration in the biomechanics and possible early development of osteoarthritis of the joint. This may affect proprioceptive properties of the foot and prevent patients from returning to preinjury activity level. Additional pearls/pitfalls and advantages/disadvantages of this technique are described in Tables 1 and 2, respectively. In conclusion, our technique for arthroscopic assisted, capsular reconstruction of the great toe MTPJ with all knotless suture anchors and suture tape augmentation provides major stability of the MTPJ while possibly reducing pain levels and promoting earlier return to play.

**TABLE 1:** Technical Pearls and Pitfalls of Arthroscopic Assisted Turf Toe Reconstruction With Knotless Suture Anchors and Suture Tape Augmentation

Pearls
‐ Great toe MTPJ arthroscopy allows visualization of joint and its outflow flushes out MMPs and inflammatory cytokines from the joint ‐ Noninvasive joint distraction to further open up great toe MTPJ for easier visualization and access ‐ Small insertional diameter of suture anchors and tape allow for easy access without taking up too much bone stock

Pitfalls
‐ Twists or kinks in suture can lead to premature locking of suture anchors causing loose repair of capsule and MTPJ instability ‐ If arthroscopic portals are created too distal or proximal to MTPJ, surgeon will struggle entering and visualizing joint due to its bilateral congruency ‐ Overtightening MTPJ joint capsule and soft tissues may lead to altered biomechanics

MMPs, matrix metalloproteinases; MTPJ, metatarsophalangeal joint.

**TABLE 2:** Advantages and Disadvantages of Arthroscopic Assisted Turf Toe Reconstruction With Knotless Suture Anchors and Suture Tape Augmentation

Advantages
‐ Minimal soft tissue disruption with MTPJ arthroscopy ‐ Utilization of all suture knotless suture anchors eliminate knots and reduce soft tissue irritation ‐ Aggressive postop protocol with weightbearing at 4 weeks and active great ROM at 8 weeks

Disadvantages
‐ Great MTPJ is an extremely small joint to undergo arthroscopy ‐ Meticulous triangulation by surgeon is important in order to prevent iatrogenic damage to MTPJ ‐ Pure precision is needed with drilling and anchor placement within metatarsal and proximal phalanx. Errors in placement may negatively affect biomechanics given limited bone stock

MTPJ, metatarsophalangeal joint; ROM, range of motion.

## DISCLOSURES

The author (K.D.M.) declares the following financial interests/personal relationships which may be considered as potential competing interests: K.D.M. reports relationships with Arthrex, iWalkFree, and XO Armor that includes consulting or advisory and reports a relationship with ConMed that includes royalties. The other authors (N.K.P., K.S.A., T.E.) declare that they have no known competing financial interests or personal relationships that could have appeared to influence the work reported in this article.
